# Experimental Results of Underwater Acoustic Communication with Nonlinear Frequency Modulation Waveform

**DOI:** 10.3390/s21217194

**Published:** 2021-10-29

**Authors:** Jeongha An, Hyungin Ra, Changhyun Youn, Kiman Kim

**Affiliations:** Department of Radio Communication Engineering, Korea Maritime and Ocean University, 727 Taejong-ro, Yeongdo-gu, Busan 49112, Korea; zzung706@g.kmou.ac.kr (J.A.); babavivi@g.kmou.ac.kr (H.R.); yoonch265@g.kmou.ac.kr (C.Y.)

**Keywords:** underwater acoustic communication, linear frequency modulation, nonlinear frequency modulation, generalized sinusoidal frequency modulation, ambiguity function, correlation function, multipath, Doppler shift, bit error rate

## Abstract

In this paper, we propose underwater acoustic (UWA) communications using a generalized sinusoidal frequency modulation (GSFM) waveform, which has a distinct ambiguity function (AF) and correlation function characteristic. For these reasons, it is more robust in multipath channels than the conventional chirp spread spectrum (CSS) with a linear frequency modulation (LFM) waveform. Four types of GSFM waveforms that are orthogonal to each other are applied for each symbol in the proposed method. To evaluate the performance of the proposed method, we compared the performances of the proposed method and conventional method by conducting diverse experiments: simulations, lake trials and sea trials. In the simulation results, the proposed method shows better performance than the conventional method. The lake trial was conducted with a distance of 300~400 m between the transmitter and receiver. As a result of the experiment, the average bit error rate (BER) of the proposed method is $3.52\times 10^{-2}$ and that of the conventional method is $3.52\times 10^{-1}$, which shows that the proposed method is superior to the conventional method. The sea trial was conducted at a distance of approximately 20 km between the transmitter and receiver at a depth of 1500 m, and the receiver was composed of 16 vertical line arrays (VLAs) with a hydrophone. The proposed method had a BER of $0.3\times 10^{-2}$ in one channel and was error free in the other.

## 1. Introduction

In recent years, underwater acoustic (UWA) communication has received much attention, with numerous applications emerging in environmental monitoring, ocean exploration, and military missions [1,2,3]. However, the UWA channel fluctuates and causes a time-varying multipath, which may result in intersymbol interference (ISI) and Doppler shifts and spreads [4,5].

Recently, several commercial UWA modems have already been introduced. In the past, the Teledyne Benthos ATM-886 model had a baud rate of 360 bps, and it has 1/2 rate convolution coding, a multipath guard period, multiple frequency shift keying (MFSK), and phase shift keying (PSK) modulation schemes [6]. LinkQuest’s SoundLink UWA modem uses broadband acoustic spread spectrum technology. The data rate achieved is up to 38,400 baud [7]. AquaSeNT provides a UWA communication modem using orthogonal frequency division multiplexing (OFDM) technology. These modems can operate in two modes: command mode and data mode [8]. DSPComm Aquacomm Gen2 modems can now operate below the noise floor at a signal-to-noise ratio (SNR) of −10 dB to −15 dB with +/−10 knot Doppler compensation in highly reflective and noisy environments. It is available in 100 bps to 1000 bps by the direct sequence spread spectrum (DSSS)/OFDM. The possible distance was tested to 8 km [9]. The Micron modem working frequency band is 20~28 kHz for the chirp spread spectrum (CSS). The data rate is 40 bps, and the micron data modem provides multipath noise rejection and compact size, with low error rates and 500 m and 150 m horizontal and vertical ranges [10]. The EvoLogics underwater communication modem is based on sweep spread technology, which provides full duplex communication. The modem has data rates up to 13.9 kbps over a 3500 m range with a frequency of 18~34 kHz [11].

The spread spectrum technique is robust to underwater wireless channel distortions; for this reason, it has been widely used in UWA communications [12]. For example, there is the frequency hopping spread spectrum (FHSS), the DSSS, and the CSS method. The CSS compensates for the drawbacks of other spread spectrum techniques and uses a wide bandwidth against various frequency selective fading; thus, it is widely used. In general, the conventional CSS method consists of linear frequency modulation (LFM) waveforms. Low sidelobe levels can be achieved without SNR loss by employing a nonlinear frequency modulation (NLFM) waveform [13].

There are many types of NLFM, such as sinusoidal frequency modulation (SFM), in which the instantaneous frequency (IF) function has a sinusoidal form [14]. However, it contains many high sidelobes in the ACF due to the periodicity of the IF. For this reason, generalized sinusoidal frequency modulation (GSFM) was suggested to eliminate the drawbacks of the SFM [15,16]. The GSFM waveform is a generalized form of the SFM waveform and uses a variable exponent parameter. Unlike the SFM waveform, the broadband ambiguity function (AF) of this variant GSFM waveform has a distinct mainlobe centered at the origin without a peak sidelobe, and the AF is very similar to a thumbtack. Designing GSFM waveforms with different parameter values (determined by the user) can produce a family of waveforms that occupy the same band of frequencies and are nearly orthogonal to each other [15]. For this reason, the GSFM waveform was recently researched in active sonar by Hague and Buck [16]. The integrated waveform based on the GSFM waveform was researched for continuous active sonar detection and communication, which was called GSFM-com [17]. That method was modulated by multiplying the baseband binary pulse amplitude modulation signal with the original GSFM signal. Therefore, it must consider phase synchronization, and the configuration of the receiver becomes complicated. In [18], a UWA communication using two types of GSFM signals was proposed.

In this paper, we propose a method with a GSFM waveform in UWA communication using four different types of waveforms: forward type, reverse-time type, forward-time/flipped-frequency type and reverse-time/flipped-frequency type. For waveforms that have near orthogonality relative to each other, the waveform minimizes the ISI by sharing public bandwidth, which means maximizing the time-bandwidth product. There is a difference from the conventional CSS method in symbols representing using multiple GSFM waveforms. Its characteristics are demonstrated using the AF and correlation in this paper. Using this orthogonality of each waveform and distinct mainlobe regions in the AF and ACF, we expect robustness in the ISI-generated multipath channel. We design the receiver, which consists of the bank of the matched filter (MF). To demonstrate the performance of the CSS method by a GSFM waveform, we evaluate the performance through an experimental comparison with the conventional CSS via a simulation, lake trial and sea trial. In this paper, the conventional CSS method is divided into two bands to match the data rate and time bandwidth product with the GSFM method. In the simulation, we consider multipath and additive white Gaussian noise (AWGN) channels, and the multipath is programmed by VirTEX (Virtual Time series Experiment), which is modeled by the Bellhop ray tracing code [19]. VirTEX was designed to model the propagation through the underwater sound channel of a known time series transmitted from a hypothetical source. The lake trial was conducted with a distance of 300~400 m between the transmitter and receiver. The sea trial was conducted at a distance of approximately 20 km between the transmitter and receiver and at a depth of 1500 m. According to [20], the conducted range of communication can be regarded as long-range communication. From the results of various experiments, this paper demonstrates the performance of the proposed method in UWA communication.

The rest of this research article proceeds as follows. Section 2 introduces the GSFM waveform and the proposed method. Section 3 simulates the proposed method and conventional method and demonstrates the comparison according to the SNRs. Section 4 demonstrates the performance of the proposed method using a lake trial and sea trial, and the results are given. Section 5 concludes the paper.

## 2. UWA Communication by Generalized Sinusoidal Frequency Modulation

The GSFM, which has a generalized form of SFM, is suggested for robust UWA communication. The GSFM waveform has much-lower-range sidelobes and maintains the mainlobe width without using the tapering function in the ACF compared with the LFM waveform.

A. Signal Design for UWA Communication

The waveform signal s(t) and IF function f(t) are expressed as shown in Equations (1) and (2), respectively [16,17].

(1) $$s\left(t\right)=Ke^{j\varphi \left(t\right)}e^{j2\pi f_{c}t},$$

(2) $$f\left(t\right)=\frac{1}{2\pi }\frac{\partial \varphi \left(t\right)}{\partial t}+f_{c}.\;$$

K in Equation (1) is the normalizing factor; $f_{c}$ is the carrier frequency; and φ(t) is the instantaneous phase (IP) of the signal.

$\varphi_{G}\left(t\right)$ and $f_{G}\left(t\right)$ are the IP and IF functions of the GSFM, respectively, and their expressions are as shown in Equations (3) and (4).

(3) $$\varphi_{G}\left(t\right)=\frac{\beta }{t^{\rho -1}}\sin\frac{\left(2\pi \alpha t^{\rho }\right)}{\rho },$$

(4) $$f_{G}\left(t\right)=\beta \left[\alpha \cos\left(\frac{2\pi \alpha t^{\rho }}{\rho }\right)-\frac{\left(\rho -1\right)}{2\pi t^{\rho }}\sin\left(\frac{2\pi \alpha t^{\rho }}{\rho }\right)\right].$$

ρ is a variable exponent parameter that can be used to adjust the asymmetric IF function of the waveform. β is a modulation index and α is the frequency modulation term.

**Figure 1:**
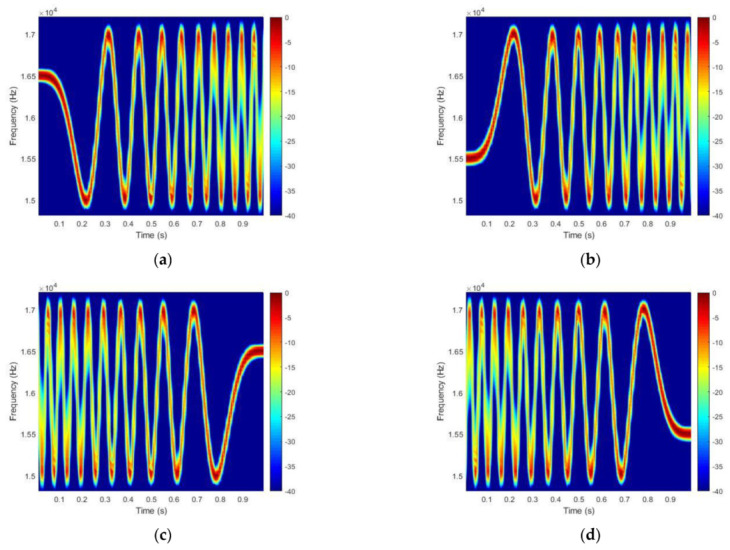
Four types of GSFM waveform spectrograms: (**a**) forward type, (**b**) forward-time/flipped-frequency, (**c**) reverse time, and (**d**) reverse-time/flipped-frequency.

In using the same index ρ=2, the waveform of the GSFM can be used to generate various types of waveforms by reversing the time and frequency domains. In this paper, we use 4 types of waveforms, which are represented in the spectrograms of Figure 1. In Figure 1, (a) represents the forward type, (b) represents the flipped frequency of type (a) and is the same as the modulated cosine function, (c) is the reverse time variant of type (a), and (d) has reversed time and a flipped frequency compared with type (a).

In this case, we assume that $f_{c}$ is 16 kHz, the bandwidth is 2 kHz and the waveform length T is 1 s. In Figure 1, β=50, and α=20 is the same as $\alpha =C/T^{\rho }$, which determines the number of cycles C=10. The four types of GSFM waveforms are orthogonal to each other. 

We expect the UWA communication performance to be more effective as a result of using the orthogonal characteristic of the GSFM AFs than that of the conventional LFM. In this paper, we use Equation (5), which is expressed as

(5) $$s_{i}\left(t\right)=\{\begin{array}{c} s_{0}\left(t\right),\;\;if\;b_{2n-1}b_{2n}=00\\ s_{1}\left(t\right),\;\;if\;b_{2n-1}b_{2n}=01\\ s_{2}\left(t\right),\;\;if\;b_{2n-1}b_{2n}=10\\ s_{3}\left(t\right),\;\;if\;b_{2n-1}b_{2n}=11 \end{array},\;\left(n-1\right)T\leq t<nT.$$

Here, $s_{0}\left(t\right)$ is the forward-type waveform, $s_{1}\left(t\right)$ is the forward-time/flipped-frequency-type waveform, $s_{2}\left(t\right)$ is the reverse-time-type waveform and $s_{3}\left(t\right)$ is the reverse-time/flipped-frequency-type waveform of the GSFM. $b_{n}$ is the *n*-th bit sequence, T is the length of the symbol, and each symbol represents a 2-bit sequence.

**Figure 2:**
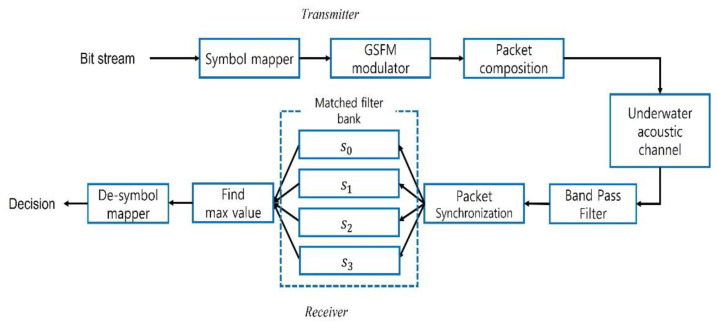
Block diagram of UWA communication by using GSFM waveform.

The block diagram of the proposed method is shown in Figure 2. The received signal that passes through a channel leaves only the band energy using a band pass filter and uses preamble for fine synchronization of the data packet. Finally, the outputs of each matched filter are compared to find the maximum value.

B. Orthogonality

The orthogonality between the waveforms representing the symbol plays an important role in the theoretical background of communication performance. The orthogonality of these waveforms determines the performance of the communication system. To determine the relation of each waveform, the AF is used, and its expression is shown in Equation (6).

(6) $$\chi_{i,j}\left(\tau ,\eta \right)=\sqrt{\eta }\int_{-\infty }^{\infty }s_{i}\left(t\right)s_{j}^{\prime }\left(\eta \left(t+\tau \right)\right)dt.$$

η is the Doppler scaling factor, and τ is the time delay.

**Figure 3:**
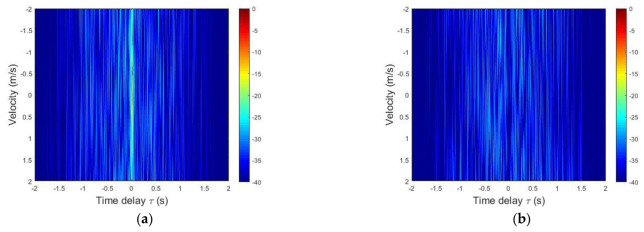

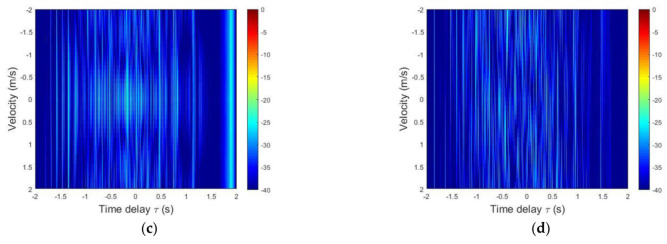
(**a**) Auto-AF of the FT, (**b**) cross-AF of the FT/FF and FT, (**c**) cross-AF of the RT and FT and (**d**) cross-AF of the RT/FF and FT.

Figure 3a represents the auto-AF of the FT, and Figure 3b–d represent the cross-AF of the FT/FF, RT and RT/FF with FT, respectively. In the auto-AF and the cross-AF of Figure 3, the time delay τ range is −2 s≤τ≤2 s, and the Doppler scaling factor η range is −2 m/s≤η≤ 2 m/s. In Figure 3a, the auto-AF has a high main level and low sidelobe level; on the other hand, even when using the same bandwidth and parameter, the cross-AF has a low correlation level. 

Furthermore, the orthogonality of the four types of GSFM waveforms can also be represented in the correlation function. To determine the correlation of each waveform, we use Equation (7) to Equation (9). Equation (7) represents the basic definition of the correlation function. By the Wiener-Khintchine Theorem, Equation (7) can be represented as Equations (8) and (9).

(7) $$R_{ij}\left(\tau \right)=\int_{-\infty }^{\infty }s_{i}\left(t\right)s_{j}\left(t+\tau \right)dt,\;$$

(8) $$S_{i}\left(f\right)=\overset{\infty }{\underset{t=-\infty }{\sum }}s_{i}\left(t\right)e^{-i\omega t}dt,\;$$

(9) $$R_{ij}\left(\tau \right)=F^{-1}\left[S_{i}\left(f\right)S_{j}^{*}\left(f\right)\right].\;$$

$F^{-1}\left[\cdot \right]$ is the inverse Fourier transform of [·], and $S_{i}\left(f\right)$ is the spectrum of $s_{i}\left(t\right)$. In Equation (7), the spectrum of the GSFM waveform is represented by Equation (10) [16].

(10) $$S_{GSFM}\left(f\right)=\left|\sqrt{T}\overset{\infty }{\underset{n=-\infty }{\sum }}J_{n}^{1:\infty }\left\{\frac{\tilde{a_{m}}\Delta fT}{2};\frac{\tilde{b_{m}}\Delta fT}{2}\right\}\times \sin c\left[\pi T\left(f-f_{c}+\frac{a_{0}\Delta f}{4}-\frac{n}{T}\right)\right]\right|.$$

**Figure 4:**
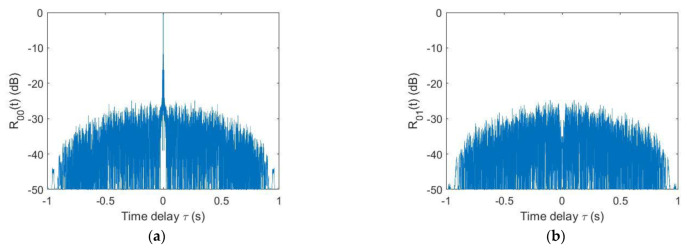

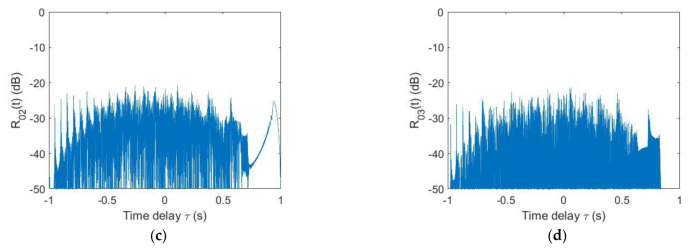
The orthogonality between waveforms, (**a**) Autocorrelation of the FT, (**b**) cross-correlation of the FT/FF and FT, (**c**) cross-correlation of the RT and FT and (**d**) cross-correlation of the RT/FF and FT.

Figure 4 represents the correlation of the FT. Figure 4a represents the ACF, Figure 4b–d represent the cross-correlation function of the FT/FF, RT and RT/FF with FT, respectively. 

## 3. Simulation Results

This simulation was compared with that of the conventional CSS method to demonstrate the performance of the proposed method, and the simulation considered two channel characteristics: multipath propagation and AWGN channels. To match the data rate, the CSS method is divided into 2 bands that also match the time bandwidth product with the proposed GSFM method. The simulation parameters are presented in Table 1. 

**Figure 5:**
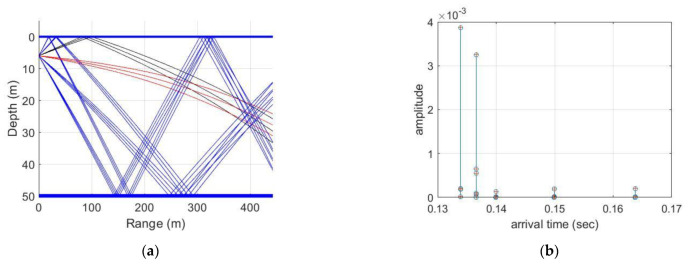
Simulation channel using the VirTEX simulator: (**a**) Ray tracing, (**b**) Channel impulse response.

**Figure 6:**
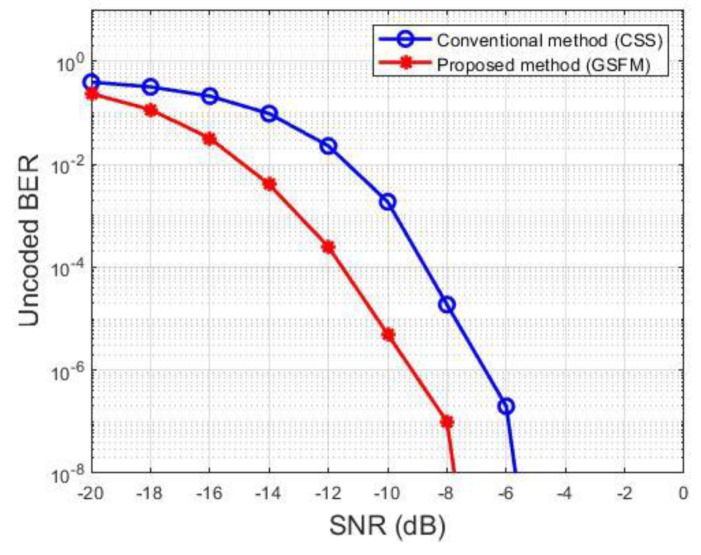
BER performance.

In the simulation, we used the VirTEX simulator with the Bellhop model for a UWA multipath channel. The simulation channel is represented in Figure 5 and assumes that the depths of the transmitter and receiver are approximately 5 m and 25 m, respectively, the water depth is 50 m and the distance between the transceivers is 400 m. In Figure 5a, the red lines represent the 1st path (direct path), the black lines represent the 2nd path and the blue lines represent the 3rd path. In this channel, Figure 5b represents the channel impulse response and represents the received signal’s arrival time depending on each path. No channel coding technique was applied to the data for simulation. The simulation result is represented in Figure 6, which shows the difference in performance according to the SNR.

In Figure 6, the horizontal axis represents the scale of the SNR, and the vertical axis represents the uncoded bit error rate (BER). It is analyzed according to the SNR level in the multipath channel. The red line represents the GSFM, which is the proposed method, and the blue line represents the conventional CSS method. We could confirm that the proposed methods have a better BER performance than the conventional CSS method. In the figure, it can be seen that the proposed method has an average gain of approximately 2~3 dB compared with the conventional method. In particular, the performance of the proposed method is relatively good in an environment with a low SNR.

## 4. Experimental Results

In this section, we verify the simulation result using two experiments: a lake trial and a sea trial. As in the simulation, each experiment compared the performance of the conventional CSS method and the proposed method.

### 4.1. Lake Trial

**Figure 7:**
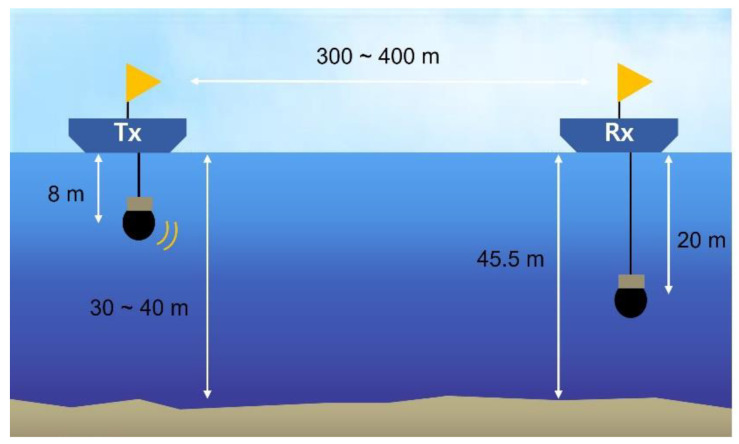
Configuration for the lake trial.

We performed a lake trial after the theoretical demonstration via the simulation. The parameters for the lake trial are presented in Table 2, and the lake experiment was constructed as shown in Figure 7. The transmitter was the Neptune Sonar D/17/BB model, and the receiver was the Teledyne Reson TC4032. At the time of the experiment, the water depth at the receiver side was approximately 45.5 m, and the receiver was located 20 m below the water surface. The transmitter was located 300~400 m away from the receiver, and the transmitter was located 8 m below the surface. The bottom topography between the transmitter and receiver was irregular. The receiver was fixed at the center of the lake, the transmitter was moved around in a barge and continuously traveled by wind and engine, and the distance between the transceivers was kept at 300~400 m.

To analyze the received signals, an estimation of the channel environment is necessary. For this reason, the underwater channel characteristics were estimated prior to this experiment. The signal used was a 128 ms LFM pulse train with a 2.5 kHz bandwidth, and it was repeated 200 times.

**Figure 8:**
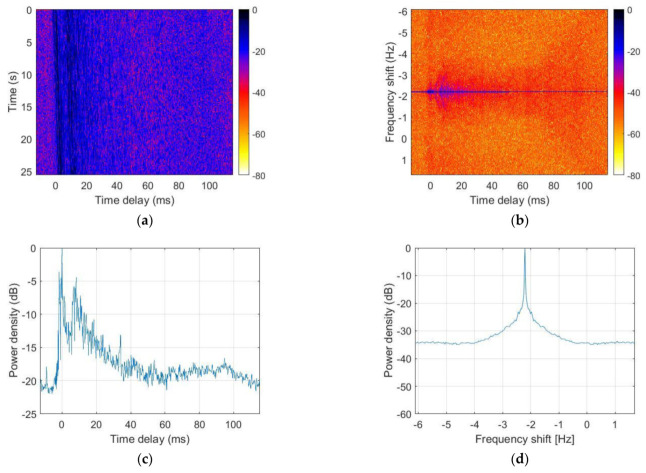
Underwater channel characteristics in the lake trial: (**a**) channel impulse response, (**b**) scattering function, (**c**) power delay profile, and (**d**) Doppler power spectrum.

Figure 8a shows the measured channel impulse response, which represents a Doppler shift and multipath. The sloped shape in the figure means that the main path signal shifts slightly with time delay over time, demonstrating a Doppler shift. This figure shows that the transceivers moved away from each other over time. In Figure 8b, we can see the scattering function. Figure 8c represents the power delay profile. Figure 8d shows the Doppler spectrum and indicates that the Doppler shift was approximately −2.21 Hz.

**Figure 9:**
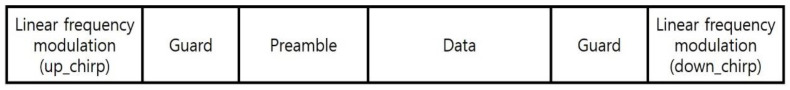
The Packet structure in the lake trial.

Figure 9 presents the packet structure used in the experiments. Both sides of the LFM waveform, which have a bandwidth of 2.5 kHz and a length of 1 s, are used to determine whether a signal exists, and the preamble, which was 511 bits modulated by binary PSK modulation for 1 kbps, is used to synchronize the signal frame accurately. The data has 336 bits of information. The lake trial was repeated 6 times.

The results of the lake trial are shown in Table 3, which represents the estimated Doppler shift frequency and uncoded BER of the proposed method and the conventional CSS method. As a result of the experiment, the average uncoded BER of the proposed method is $3.52\times 10^{-2}$ and that of the conventional method is $3.52\times 10^{-1}$, which shows that the proposed method is superior to the conventional method.

### 4.2. Sea Trial

**Figure 10:**
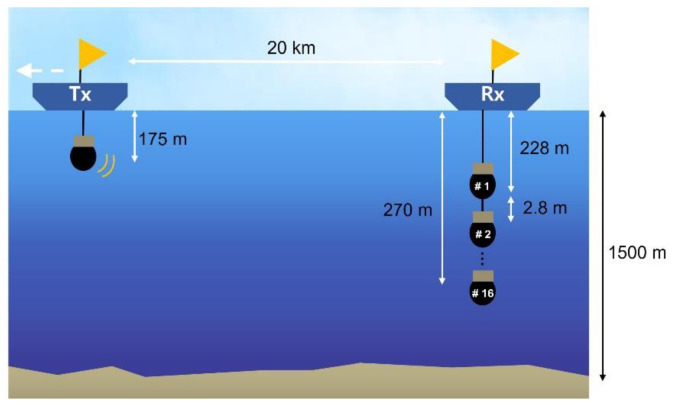
Configuration for sea trial.

In November 2020, the sea trial was conducted in the East Sea of Korea. The sea trial parameters are presented in Table 4, and the experimental configuration was constructed as shown in Figure 10. The transmitter was composed of a projector that was moored, and the depth of the projector was approximately 175 m. The transmitter was moved at a speed of 3 knots for approximately 20 km, and the transmitter moved away from the receiver. The receiver was composed of 16 vertical line arrays (VLAs) with a hydrophone interval of 2.8 m, which was located at the center of the array at a depth of 250 m. The receiver was moored on a buoy, and the water depth was approximately 1500 m at the receiver side.

**Figure 11:**
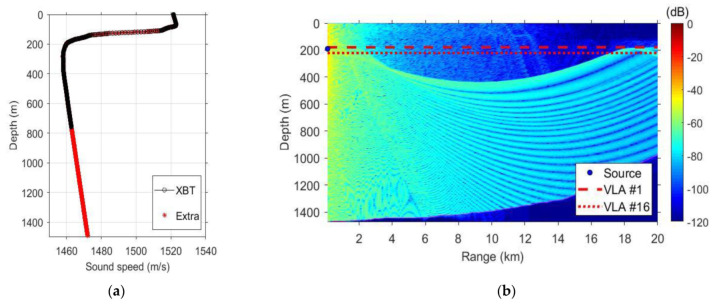
Underwater channel in the sea trial: (**a**) sound speed profile and (**b**) transmission loss.

Figure 11a represents the measured sound speed profile (SSP) at the experimental site, and Figure 11b represents the transmission loss by Bellhop modeling. The SSP was measured by using expendable bathy thermography (XBT). The XBT measures the temperature profile and computed sound velocity data. The measurable extent of the XBT used was a water depth of 750 m, and a greater depth was generated by extrapolation. Using the SSP in Figure 11a, the transmission loss is calculated in Figure 11b. Figure 11b shows that the transmission loss at the depth where the receivers are located is relatively low at a distance of approximately 20 km. Therefore, a relatively high SNR was predicted.

Before receiving signals, we measured the channel impulse response. For the measure of the channel, the signal had a length of 500 ms data composed of a 31 ms LFM pulse train with a 1500 Hz bandwidth, and it was repeated 300 times.

**Figure 12:**
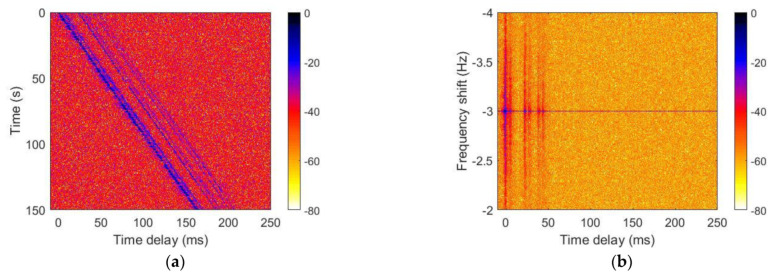

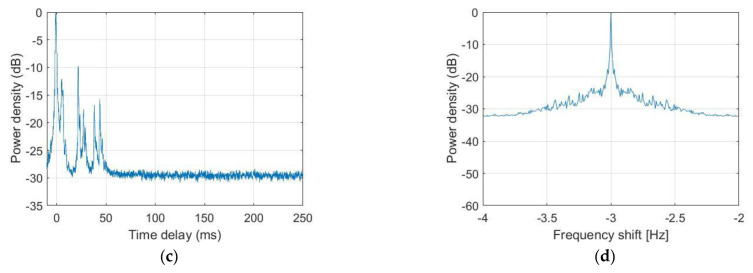
Underwater channel characteristics in the sea trial: (**a**) channel impulse response, (**b**) scattering function, (**c**) power delay profile, and (**d**) Doppler power spectrum.

**Figure 13:**
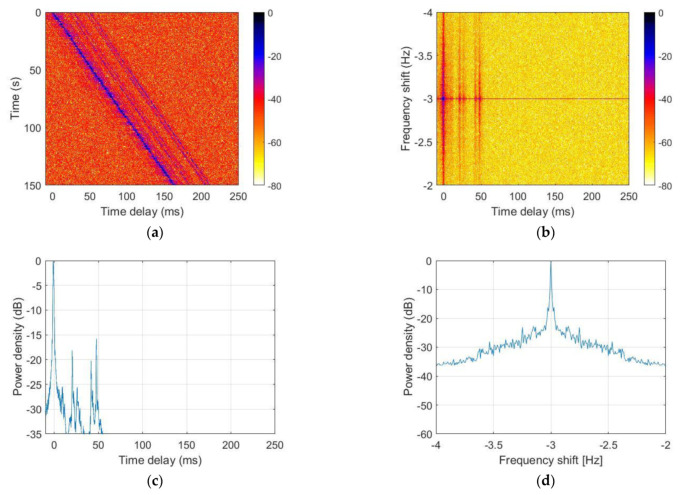
Underwater channel characteristics in the sea trial: (**a**) channel impulse response, (**b**) scattering function, (**c**) power delay profile, and (**d**) Doppler power spectrum.

Figure 12 and Figure 13 show the UWA channel characteristics estimated at the 7th and 16th receivers, respectively. The channel has diverse multipaths and a slight sloping shape when the transmitter moves away from the receiver. Figure 12 and Figure 13b,d show that the Doppler shift was approximately −3 Hz.

**Figure 14:**
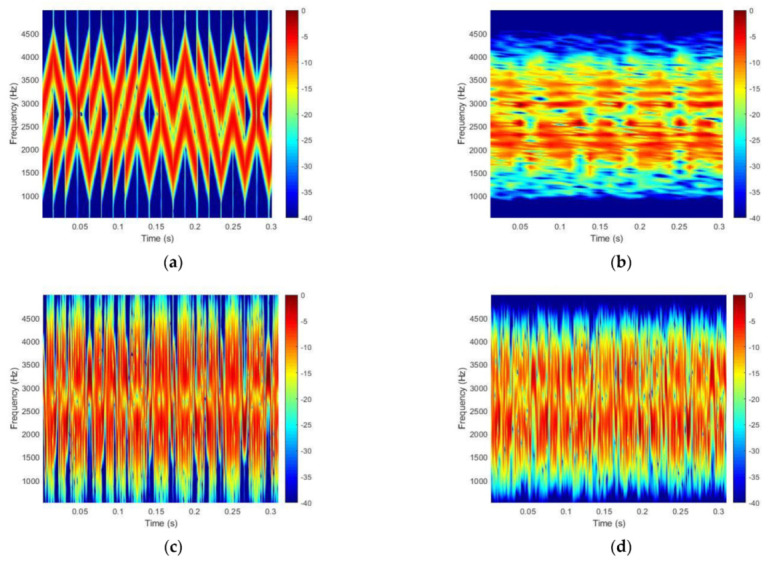
The spectrogram of the data: (**a**) the transmitted signal, (**b**) the received signal of the conventional CSS method, (**c**) the transmitted signal and (**d**) the received signal of the proposed GSFM method.

Figure 14 represents the spectrogram of the signals in the sea trial. Figure 14a,b represents the part of the transmitted CSS signal and the received CSS signal, and Figure 14c,d represents the part of the transmitted GSFM signal and the received GSFM signal, respectively. The channel coding technique was not applied. As a result of the experiment, the conventional CSS method is error free for all channels. The uncoded BER of the proposed GSFM method is $0.3\times 10^{-2}$ for one channel, and the other is error free. However, this is within the range that can be completely decoded if the channel coding method is applied. As mentioned earlier, the SNR of the received signal was relatively high because the transmission loss was low at the experimental point, and as a result, the performances of the two methods were not significantly different. 

## 5. Conclusions

In this paper, we proposed UWA communication using GSFM waveforms, which offers advantageous modulation in a fluctuating UWA channel, and the proposed method’s performance was demonstrated by a comparison with that of the conventional CSS method. According to the AF and ACF, we demonstrated that the GSFM waveform has a low sidelobe level and a distinct mainlobe level simultaneously, which has better performance in signal interference. Regarding the reliability of the proposed method, experiments were conducted using three kinds of methods: a simulation that considered the multipath propagation and AWGN channel, a lake trial and a sea trial. In this paper, multiple GSFM waveforms orthogonal to each other are used to transmit data in UWA communication. This is what sets it apart from past studies and is our original contribution on this topic.

The performance of UWA communication is not simply proportional to the trans-mission distance. It is affected by many factors, such as the SSP, the placement of the transceiver, the sea state and the geometry of the channel. In a lake environment, multipath propagation is very severe, affecting the UWA communication performance. This is also shown in the experimental results presented in this paper. The trial environment was a deep sea, and since it is an open space, it was less affected by such multipath propagation. As a result, the proposed method is more robust in UWA communication with multipath propagation and AWGN channels than the conventional CSS method. The Doppler channel was not taken into account in the simulation, but the acquired data from the lake and sea trial contained Doppler shifts of several Hz. If it is to be applied in a severe Doppler channel, a Doppler shift frequency estimation and a modified correlation function will probably be added. There is a need for research on performance verification and improvement in the Doppler channel in future research.

## Figures and Tables

**Table 1:** Simulation parameters.

Parameter	Value
Sampling frequency	192 kHz
Carrier frequency	16 kHz
Data rate	100 bps
Bandwidth	2.5 kHz
SNR	−20~0 dB

**Table 2:** Parameters for the lake trial.

Parameters	Value
Sampling frequency	192 kHz
Carrier frequency	16 kHz
Data rate	100 bps
Bandwidth	2.5 kHz
Range between projector and hydrophone	300~400 m
Projector depth	8 m
Hydrophone depth	20 m
Water depth at projector side	30~40 m
Water depth at hydrophone side	45.5 m
Projector	Neptune Sonar D/17/BB
Hydrophone	Teledyne Reson TC4032

**Table 3:** Uncoded BER in the lake trial.

No.	Doppler (Hz)	Proposed Method (GSFM)	Conventional Method (CSS)
1.	−2.15~−1.32	0	$3.10\times 10^{-1}$
2.	−1.33~−1.3	$1.19\times 10^{-2}$	$4.61\times 10^{-1}$
3.	−4.29~−4.59	$1.07\times 10^{-1}$	$3.69\times 10^{-1}$
4.	1.33~ 4.91	$2.08\times 10^{-2}$	$3.87\times 10^{-1}$
5.	−1.05~−3.94	$4.17\times 10^{-2}$	$3.93\times 10^{-1}$
6.	−4.99~−0.38	$2.98\times 10^{-2}$	$1.91\times 10^{-1}$
Average		$3.52\times 10^{-2}$	$3.52\times 10^{-1}$

**Table 4:** Parameters for the sea trial.

Parameter	Value
Sampling frequency	16,384 Hz
Carrier frequency	2750 Hz
Data rate	128 bps
Bandwidth	2500 Hz
Range between projector and hydrophone	Approximately 20 km
Projector depth	Approximately 175 m
Hydrophone depth	228~270 m (2.8 m interval)
Water depth at projector side	1500 m
Projector	Neptune Sonar T-161
Hydrophones	16-VLA

## Data Availability

Not applicable.
